# GALLBLADDER CANCER AS INCIDENTAL FINDING IN TWO STAGE RESOLUTION OF GALLSTONE ILEUS

**DOI:** 10.1590/S0102-67202015000300020

**Published:** 2015

**Authors:** César Muñoz CASTRO, Héctor Losada MORALES, Marcelo Santelices BAEZA

**Affiliations:** 1 Departamento de Cirugía, Universidad Católica del Maule, Talca; 2 Departamento de Cirugía, Universidad de la Frontera, Temuco; 3 Servicio de Cirugía, Hospital Regional de Talca, Talca, Chile

## INTRODUCTION

Gallstone ileus (GI) is a rare complication of biliary pathology when a bile stone from gallbladder or exceptionally from the main bile duct, cause an obstruction of the intestinal lumen^10^. Gallstone ileus incidence has remain constant through the years in 0,9 cases for 100.000 admissions/year^6^. 

The diagnosis is usually difficult because of the abscense of specific symptoms, and sometimes by the partial remission of them during the migration of the bile stone through the intestinal lumen. This situation usually delays the consultation until there is greater compromise of the patient´s general condition. The imaging studies, either simple radiology, ultrasound or computarized axial tomography of the abdomen are useful in the early diagnosis^1^. The initial treatment for GI is the reanimation and stabilization of the electrolite imbalance that might present on this patients and later perform the surgical resolution of the bowel obstruction.

The objective of this report is to present the finding of a gallbladder cancer in the two-stage resolution of a GI and discuss some aspects about the treatment of this disease.

## CASE REPORT

**FIGURE 1:**
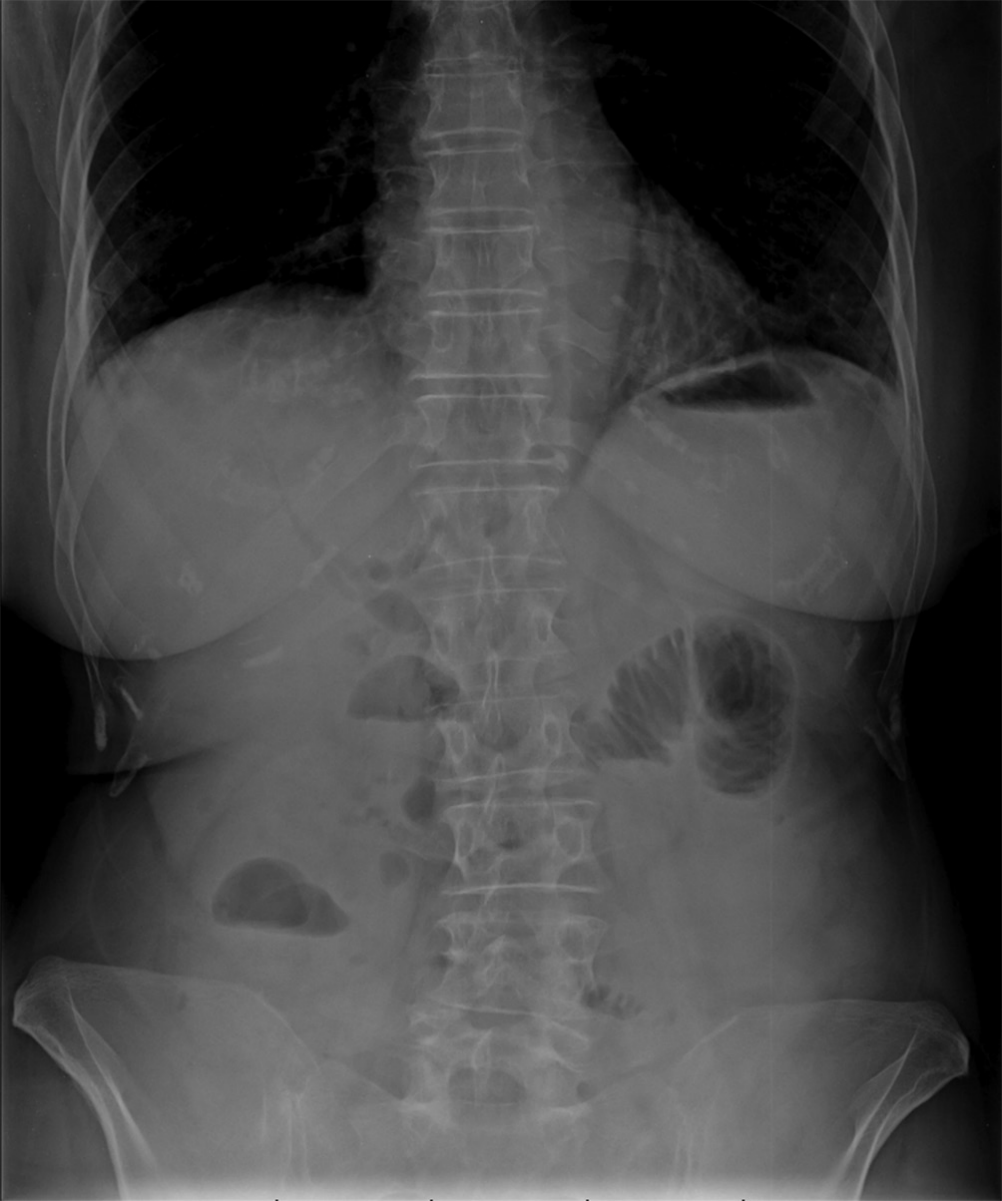
Plain abdominal x-ray showing small bowel dilatation and pneumobilia

Seventy-two years old female, with previous coronary heart disease, that look for medical assistance due to epigastric and right upper quadrant abdominal pain plus vomiting of a few days of evolution. Her physical exam showed tenderness on the right upper quadrant, without palpable mass. The laboratory test resulted with leukocytosis of 14900 cel/mm^3^, C reactive protein of 104 mg/dl and all others were normal. A plain abdominal X-ray (Figure 1) and abdominal ultrasound were performed, and showed pneumobilia associated with an ovoid image in the mid jejunum with a change in the caliber of the bowel. With the diagnosis of GI a exploratory laparotomy was performed, with findings of two big bile stones at the mid jejunum. A longitudinal enterotomy was performed, with enterolithotomy and closure in one plane of suture. The patient evolved without complications and was discharched on the fifth day after the surgery. 

One month after the surgery, the patient remained asymptomatic. A new ultrasound was performed that showed a scleroatrophic gallbladder without evidence of cholelythiasis and a common bile duct of 5 mm. The patient rejects the surgery for cholecystectomy and closure of the bile fistulae, and was maintained in ambulatory controls. 

Two years after the surgery, she had episodes of colic abdominal pain, associated with jaundice and fluctuant choluria. An abdominal ultrasound and a cholangiomagnetic resonance revealed alithiasic schleroatrophic gallbladder, with dilatation of the extrahepatic bile duct and choledocolythiasis. With these findings, exploratory laparotomy was decided for cholecistectomy and exploration of the choledocus. In the surgery is found a subhepatic adherencial process with a schleroatrophic gallbladder, persistency of an active cholecystoduodenal fistulae and dilatation of the extrahepatic bile duct of 12 mm. A cholecystectomy with resection in block of the fistulous tract with the compromised duodenum was performed, with exploration of the common bile duct extracting various pigmentary bile stones, choledocostomy with Kehr catheter nº 16 and closure of the duodenum in one plane. The patient evolved without signs of complication and was discharged at the third day after surgery.

**FIGURE 2:**
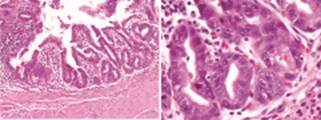
Epithelial neoplasm with tubulo-papilar pattern, cells with anisocaryosis, hyperchromasis, ocasionaly prominent nucleolus and athypical mytosis

Histopathology of the surgical specimen was pT1b (Figure 2). After 24 months of follow up the patient remained asymptomatic without signs of local or systemic recurrence. 

## DISCUSSION

The GI accounts for 1-4% of intestinal obstruction and can reach up to more than 20% when are considered only patients over 60 years^5^. The obstruction occurs at any level of the gastrointestinal tract, but the more frequent site is distal ileum (>60%)^8^. The gastroduodenal obstructions due to biliary stone can be presented as gastric retention syndrome or Bouveret´s syndrome^4^. 

The clinical diagnosis is not easy, because of the slow and intermitent evolution of the disease; in this stage image exams can be helpful. The plain abdominal x-ray can show presence of pneumobilia, intestinal dilation with hydroaerial levels and radiologic evidence of ectopic stone, or Rigler´s triad. The abdominal ultrasound can show presence of pneumobilia, confirm the presence of gallstones and ocasionally demonstrate the presence of a stone either in bile digestive fistulae or in intestinal lumen. Abdominal computarized tomography has proven to be usefull in the preoperative diagnosis and allows to characterize the patient´s clinical status, also the magnitude of the obstruction.

For surgical treatment several alternatives has been proposed. The first, corresponds to the enterolithotomy or intestinal resection as only treatment without other intervention. This treatment option usually is performed in patients with surgical high risk or in whom the life spam are lower because of their comorbidities^7^. The second, is called "two-steps resolution"; this modality contemplate an enterotomy or intestinal resection as first step, and 4-6 weeks after the resolution of the GI, the cholecistectomy is performed with repair of the bile digestive fistulae^9^. The third, is a "one-time" surgery that contemplate a enterolithotomy or intestinal resection, cholecistectomy and repair of the bile digestive fistulae in the same operative act; however, this modality is associated with higher morbidity and is recommended for younger patients, without commorbidities and with low surgical risk^8^. The laparoscopic surgery is also an option of treatment that has proven to be effective for the GI with different alternatives previously discussed^2^ ^6^. There are reports of spontaneous resolution and evacuation of GI with conservative non-surgical treatment; but, it evolve with worse outcomes in terms of morbidity and mortality in comparation with the surgical treatment^3^. 

The patients treated with two-steps surgery can reject the second intervention if they do not present symptoms after four weeks, or some surgeons might obviate this procedure in elderly patients with abscense of residual lithiasic disease in control ultrasonography. We believe that the risk of gallbladder cancer should be considered in these patients during their evolution, because, even though infrequent, this population has higher risk than the population in general with cholecystolithiasis alone^11^.
